# Incidence, risk factors and clinical outcomes of septic acute renal injury in cancer patients with sepsis admitted to the ICU: A retrospective study

**DOI:** 10.3389/fmed.2022.1015735

**Published:** 2022-12-14

**Authors:** Yong Yang, Jun Dong, Xiaojie Chen, Renxiong Chen, Hongzhi Wang

**Affiliations:** Key Laboratory of Carcinogenesis and Translational Research (Ministry of Education/Beijing), Department of Critical Care Medicine, Peking University Cancer Hospital and Institute, Beijing, China

**Keywords:** cancer, sepsis, acute kidney injury, risk factor, outcome

## Abstract

**Background:**

The purpose of this study was to clarify the incidence, risk factors, and clinical outcomes of septic acute kidney injury (AKI) in cancer patients with sepsis admitted to the intensive care unit (ICU).

**Methods:**

A total of 356 cancer patients admitted to the ICU due to sepsis from January 2016 to October 2021 were analyzed retrospectively. According to the incidence of septic AKI, all patients were divided into the non-AKI group (*n* = 279) and the AKI group (*n* = 77). The clinical data after ICU admission were compared between the above two groups, and the risk factors and the clinical outcomes of septic AKI in the ICU were identified.

**Results:**

The incidence of septic AKI in all patients was 21.6% (77/356). LASSO regression and logistic regression all showed that lactate, sequential organ failure assessment (SOFA) score and septic shock were closely related to the occurrence of septic AKI. In terms of clinical outcomes after ICU admission, the rate of mechanical ventilation (MV) and continuous renal replacement therapy (CRRT), MV time, hospitalization time and 28-day mortality in the ICU were significantly higher in the septic AKI group than in the non-septic AKI group. Among the three subgroups of septic AKI (AKI combined with septic shock, septic cardiac dysfunction or acute respiratory failure), the mortality of patients in the subgroup of AKI combined with septic shock was significantly higher than others. CRRT has no significant effect on the short-term outcome of these patients.

**Conclusion:**

Lactate level, SOFA score and septic shock were closely related to the occurrence of septic AKI in the ICU. The clinical outcomes within 28 days after ICU admission of cancer patients with septic AKI were worse than those without septic AKI. The short-term outcome was worse in patients with septic AKI complicated with septic shock. CRRT does not have any significant effect on the short-term prognosis of cancer patients with septic AKI in the ICU.

## Introduction

Acute kidney injury is considered as one of the serious comorbidities in critically ill patients. AKI may have higher short-term and long-term mortality, and the use of medical resources is considerably increased. AKI is characterized by a sudden decrease in glomerular filtration rate (GFR), resulting in the accumulation of nitrogenous waste and the inability to maintain the homeostasis of body fluids and electrolytes (1). Although there is not any clear causal relationship between AKI and chronic kidney disease (CKD), the AKI non-intervention group may increase the risk of CKD (2). Patients with AKI are the most likely to suffer from accelerated loss of renal function and progress to CKD than patients without AKI with all else being equal (3). CRRT is an effective treatment for AKI, but it does not reduce long-term mortality of AKI or the risk of CKD (4). Even if AKI patients return to normal kidney function after discharge from the hospital, there is still a risk of adverse kidney events for up to 10 years (5). In addition, a meta-analysis suggests that the duration of AKI is independently related to long-term mortality, cardiovascular events and the development incident CKD of stage 3 (6). Considering the above situation, AKI should be given full attention and early disposal.

The most common cause of AKI in critically ill patients is sepsis. Cohort studies indicate that the incidence of septic AKI ranges from 19 to 48%, while the mortality of patients with septic AKI fluctuates from 22 to 70% (7, 8). The pathophysiology of septic AKI is still not fully appreciated. Traditionally, it is believed that septic AKI is mainly caused by global renal ischemia and hypoperfusion, septic endotoxin-mediated cell damage, and renal tubular necrosis (9). However, other studies suggest that septic AKI is a bioenergy adaptive response of the body to microcirculation dysfunction and inflammation caused by sepsis, which has no significant correlation with the existence of systemic hypoperfusion or the severity of sepsis (10–12).

Because the immune system of cancer patients with sepsis cannot cope with the initial injury, pathogen invasion emerged on the basis of malignant cell transformation. Compared with non-cancer patients, cancer patients with sepsis had a 2.5-fold higher in-hospital mortality rate due to sepsis. Cancer patients with sepsis have a worse prognosis (13, 14). Therefore, there may be a great proportion of septic AKI in cancer patients with sepsis. This retrospective study aimed at the precise population of cancer patients with sepsis to clarify the incidence, risk factors and short-term clinical outcomes of septic AKI after ICU admission to guide clinical intervention and judge prognosis.

## Materials and methods

### Participants

**FIGURE 1:**
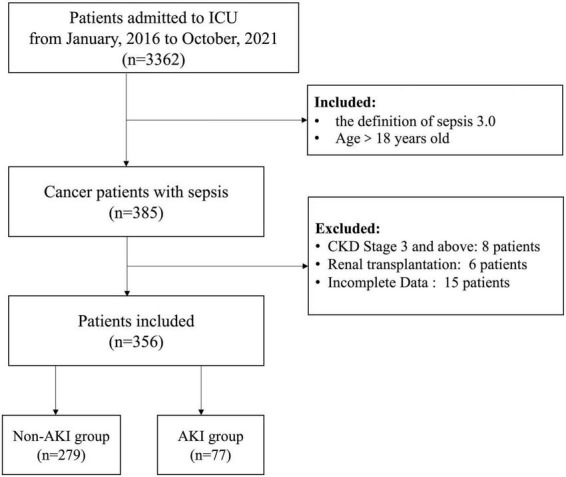
Flow chart of the research scheme.

The study complies with the Declaration of Helsinki and was approved by the Ethics Committee of Peking University Cancer Hospital(ethics approval number 2020KT33), and all patients provided written informed consent for the treatment of sepsis and related scientific research purposes A total of 356 cancer patients with sepsis were retrospectively screened out of 3,362 patients admitted to the ICU in Peking University Cancer Hospital from January 2016 to October 2021, according to the inclusion criteria. Inclusion criteria: (1). Patients with sepsis aged >18 years; (2). Diagnosis satisfying the definition of sepsis 3.0. Exclusion criteria: (1). CKD stage 3 and above; (2). After kidney transplantation; (3). Incomplete clinical data. All the included patients were divided into the non-AKI group (*n* = 279) and the AKI group (*n* = 77) in terms of the occurrence of septic AKI (Figure 1).

The diagnosis of septic AKI: (1). Clinical judgment of AKI has a positive correlation with sepsis; (2). AKI refers to the definition and diagnostic criteria from kidney disease improving global outcomes (KIDGO) in 2012.

### Data collection

Demographic characteristics and baseline data [including sex, age, body mass index (BMI), cancer types, cancer treatment, chronic diseases], infection site and etiological data, some laboratory test results within 24 h after ICU admission, the first SOFA scores, and other complications related to sepsis, including septic shock, septic cardiac dysfunction (SCD), and acute respiratory failure (ARF), were collected from all the included patients. The short-term clinical outcomes of all patients in the ICU were recorded, including mechanical ventilation (MV), continuous renal replacement therapy (CRRT), MV time, length of stay in the ICU and 28-day mortality in the ICU.

### Statistics

SPSS 26.0 (Armonk, NY: IBM) and R language (version 4.1.2, involving software packages such as *“survival,” “surviviner,” “glmnet,” “pROC”*) were used for statistical analysis. Continuous variables with normal distributions were expressed as means ± SD; otherwise, they were expressed as medians (IRQ). Categorical data were expressed as numbers (proportions). categorical variables were reported as frequency or percentage (%). Continuous variables with a normal distribution were compared by unpaired independent t test, continuous variables with a skewed distribution were compared by the Mann–Whitney U test, and the classified data were compared using the χ2 test or Fisher’s exact probability method. Logistic regression and LASSO regression were utilized to compare and screen out the significant risk factors of septic AKI. The number of septic AKI related variables of non-zero parameters was controlled by adjusting the Lambda(λ) value in the LASSO regression. 1se (the dashed line on the right side) was taken as a reference, the method of ten-fold cross-validation was utilized to obtain the minimum number of variables for the optimal model. The Kaplan–Meier method was used to analyze the short-term clinical prognosis of patients with septic AKI. The ROC curve was used to determine the predictive value of relevant risk factors for septic acute renal injury. Bivariate correlation analysis is applied to the comparison of critical variables. For all the above tests, a two-tailed *P* < 0.05 was regarded as statistically significant.

## Results

1. Occurrence of sepsis-related AKI among different cancer types: After being regrouped, septic patients with retroperitoneal cancers and urinary cancers were more likely to suffer from septic AKI (*P* = 0.002) (Table 1).

**TABLE 1:** Demographic data and characteristics of the two groups.

	Non-AKI group (*n* = 279)	AKI group (*n* = 77)	*P*-value
Sex, male	205 (73.4%)	57 (74.0%)	0.832
Age (year)	63.9 ± 9.8	61.9 ± 12.3	0.206
BMI (kg/m^2^)	22.8 ± 5.1	23.7 ± 7.0	0.195
Cancer types			0.002
Lung	29 (10.4%)	7 (9.1%)	
Digestive system	202 (72.4%)	42 (54.5%)	
Retroperitoneum	18 (6.5%)	13 (16.9%)	
Uria	4 (1.4%)	7 (9.1%)	
Bone and soft tissue	2 (0.7%)	1 (1.3%)	
Gynecology	9 (3.2%)	3 (3.9%)	
Breast	4 (1.4%)	1 (1.3%)	
Lymphoma	4 (1.4%)	1 (1.1%)	
Melanoma	3 (1.0%)	1 (1.3%)	
Others	4 (1.4%)	1 (1.1%)	
Cancer treatment			
Surgery	186 (66.7%)	47 (61.0%)	0.358
Chemotherapy	106 (38.0%)	34 (44.2%)	0.327
Radiotherapy	37 (13.3%)	11 (14.3%)	0.816
Targeted therapy	53 (19.0%)	16 (20.8%)	0.726
Immunotherapy	34 (12.2%)	12 (15.6%)	0.431
Chronic diseases			
Hypertension	63 (22.6%)	20 (26.0%)	0.533
Diabetes	49 (17.6%)	13 (16.9%)	0.889
Coronary heart disease	43 (15.4%)	7 (9.1%)	0.158
COPD	32 (11.5%)	7 (9.1%)	0.489
Cerebrovascular disease	25 (9.0%)	6 (7.8%)	0.748

AKI, acute kidney injury; BMI, body mass index; COPD, chronic obstructive pulmonary disease.

2. Comparison of variables with statistical differences between the two groups of patients: There were significant differences in creatinine, lactate, procalcitonin (PCT), brain natriuretic peptide (BNP), and SOFA scores after ICU admission between the septic AKI group and the non-septic AKI group (*P* = 0.032, *P* = 0.002, *P* = 0.001, *P* = 0.005, *P* = 0.001) (Table 2).

**TABLE 2:** Infectious data and laboratory data of the two groups after ICU admission.

	Non-AKI group (*n* = 279)	AKI group (*n* = 77)	*P*-value
Infection category			0.620
Respiratory	92 (32.9%)	21 (27.2%)	
Gastrointestinal	20 (7.1%)	8 (10.3%)	
Abdominal cavity	129 (46.2%)	39 (50.6%)	
Thoracic cavity	27 (9.7%)	6 (7.8%)	
CLABSI	3 (1.1%)	1 (1.2%)	
Genitourinary	5 (1.8%)	1 (1.3%)	
Others	3 (1.1%)	1 (1.3%)	
Organism			0.712
Gram negative	89 (31.9%)	23 (29.9%)	
Gram positive	41 (14.7%)	13 (16.8%)	
Fungi	27 (9.7%)	8 (10.4%)	
Two or more	56 (20.1%)	16 (20.8%)	
Laboratory examination			
Leukocyte (10^9^/L)	9.6 ± 5.5	8.9 ± 7.0	0.691
Neutrophil (10^9^/L)	7.6 (4.9–10.7)	6.8 (3.5–11.0)	0.442
Lymphocyte (10^9^/L)	0.5 (0.3–1.0)	0.5 (0.2–0.8)	0.420
NLR	14.9 (7.9–20.5)	13.0 (6.8–22.0)	0.918
Creatine (μmol/L)	66.3 ± 15.5	147.8 ± 73.9	0.032
Albumin (g/L)	31.9 ± 4.8	31.3 ± 3.6	0.280
Lactate (mmol/L)	2.4 ± 1.5	4.3 ± 3.5	0.002
PCT (ng/mL)	10.3 ± 23.9	39.6 ± 74.4	0.001
BNP (pg/mL)	550.7 ± 704.2	913.5 ± 1046.1	0.005
cTnI (ng/mL)	0.03 (0.01–0.14)	0.08 (0.03–0.31)	0.106
Severity of illness			
SOFA score	6.0 (5.0–9.0)	10.0 (7.0–14.0)	0.001

CLABSI, central line associated blood stream infection; NLR, neutrophil-to-lymphocyte ratio; PCT, procalcitonin; BNP, brain natriuretic peptide; cTnI, cardiac troponin I.

3. Comparison of sepsis-related complications between the two groups: Sepsis-related complications (septic shock, SCD and ARF) were more likely to occur in the septic AKI group than in the non-septic AKI group (*P* = 0.001, *P* = 0.001, *P* = 0.041) (Table 3).

**TABLE 3:** Other complications between the two groups.

Other complications	Non-AKI group (*n* = 279)	AKI group (*n* = 77)	*P*-value
Septic shock	99 (35.5%)	57 (74.0%)	0.001
SCD^#^	40/166 (24.1%)	29/61 (47.5%)	0.001
ARF	126 (45.2%)	45 (58.4%)	0.041

SCD, septic cardiac dysfunction; ARF, acute respiratory failure. ^#^227 patients (166 patients in the non-AKI group and 61 patients in the AKI group) underwent bedside echocardiography.

**FIGURE 2:**
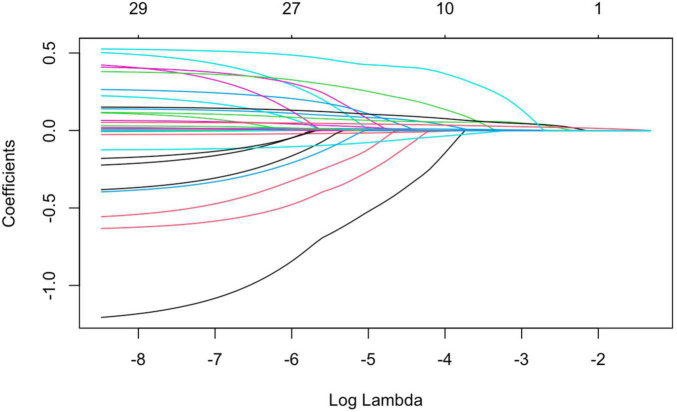
All variables were screened with LASSO regression.

**FIGURE 3:**
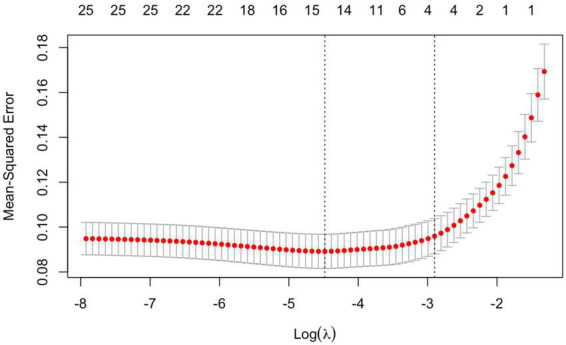
Important variables identified with ten-fold cross-validation.

4. LASSO regression was used to screen the important risk factors of septic AKI: All the variables in Tables 1–3 were screened with LASSO regression for avoiding overfitting the data in order to improve accuracy (Figures 2, 3). These important variables including lactate, SOFA score, septic shock, and PCT were strongly associated with septic AKI.

5. Independent risk factors of septic AKI were screened out by multivariate analysis of Logistic regression: Lactate, SOFA score and septic shock (variables from Tables 1, 3) were closely related to septic AKI, and these three variables were independent risk factors for septic AKI (*P* = 0.001, *P* = 0.001, *P* = 0.009) (Table 4).

**TABLE 4:** Multivariate analysis with logistic regression in the two groups.

Variables	*B*	Wald	*P*-value	OR	95% CI
Lactate	0.228	10.095	0.001	1.256	1.091–1.446
SOFA score	0.164	18.781	0.001	1.179	1.094–1.270
Septic shock	0.849	6.778	0.009	2.338	1.234–4.430

SOFA, sequential organ failure assessment.

**FIGURE 4:**
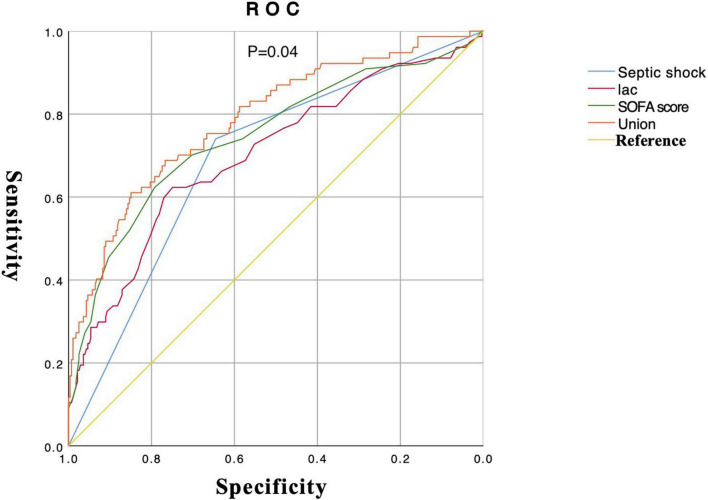
Drawing of multivariable ROC curve.

6. The drawing of ROC about important variables that affect the occurrence of septic AKI: Lactate, SOFA score, and septic shock were screened out with the intersection of Wayne diagram adopted from the combination of Lasso regression and logistic regression. The union ROC (lactate combined with SOFA score and septic shock) showed that the performance in predicting septic AKI (AUC 0.79, 95% CI 0.73–0.85) is better than the predictive performance of each variable (septic shock, AUC 0.69, 95% CI 0.63–0.76; lactate, AUC 0.70, 95% CI 0.63–0.77; SOFA sore, AUC 0.74, 95% CI 0.67–0.81) (*P* = 0.04) (Figure 4). Bivariate correlation analysis of these three variables showed that there was a positive correlation between septic shock and lactate (*P* < 0.001, *r* = 0.330), a positive correlation between septic shock and SOFA score (*P* < 0.001, *r* = 0.413), and a positive correlation between lactate and SOFA score (*P* < 0.001, *r* = 0.378).

7. Comparison of the difference on the short-term clinical outcome between two groups: In terms of short-term clinical outcomes, patients with septic AKI had higher rates of MV and CRRT, longer durations of MV-time and ICU stay-time, and higher 28-day mortality in the ICU (*P* = 0.004, *P* = 0.001, *P* = 0.006, *P* = 0.004, *P* = 0.001) (Table 5).

**TABLE 5:** Clinical outcomes with the two groups.

	Non-AKI group (*n* = 279)	AKI group (*n* = 77)	*P*-value
MV	119 (42.7%)	47 (61.0%)	0.004
CRRT	1 (0.3%)	24 (31.2%)	0.001
ICU MV-time(day)	3.0 ± 5.4	5.4 ± 6.9	0.006
ICU stay-time(day)	7.3 ± 5.1	11.7 ± 12.9	0.004
The 28-day mortality	23 (8.2%)	37 (48.1%)	0.001

MV, mechanical ventilation; CRRT, continuous renal replacement therapy; ICU, intensive care unit.

**FIGURE 5:**
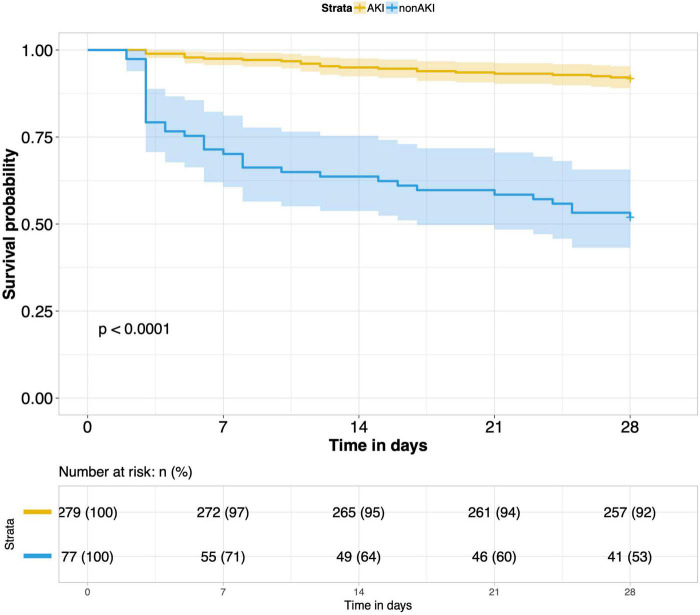
Survival analysis of septic acute kidney injury (AKI) group and non-septic AKI group.

**FIGURE 6:**
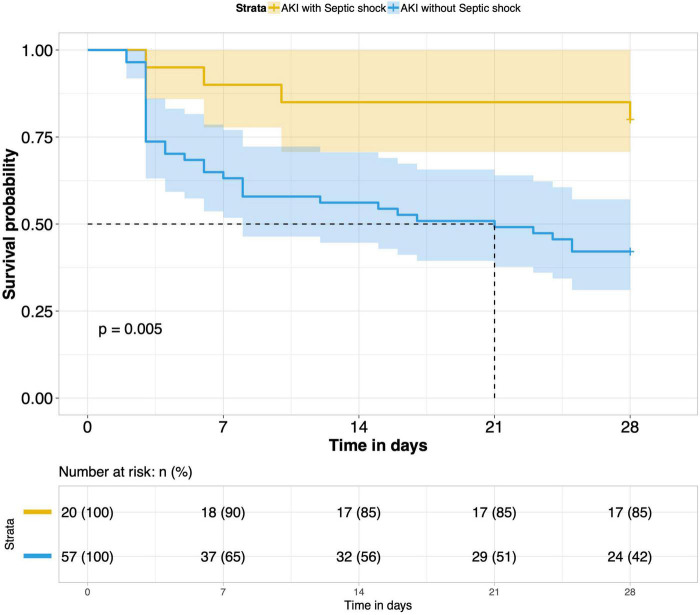
Survival analysis of AKI with septic shock group and AKI without septic shock group.

**FIGURE 7:**
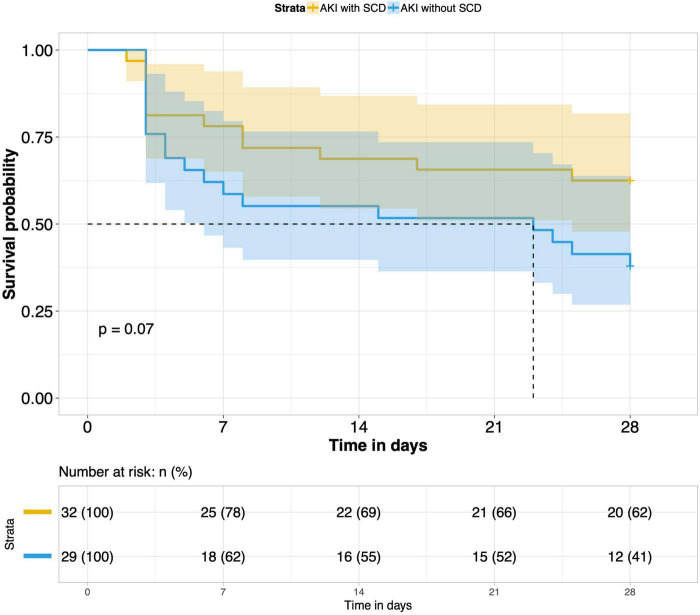
Survival analysis of AKI with SCD group and AKI without SCD group (61 of 77 patients underwent bedside echocardiography).

**FIGURE 8:**
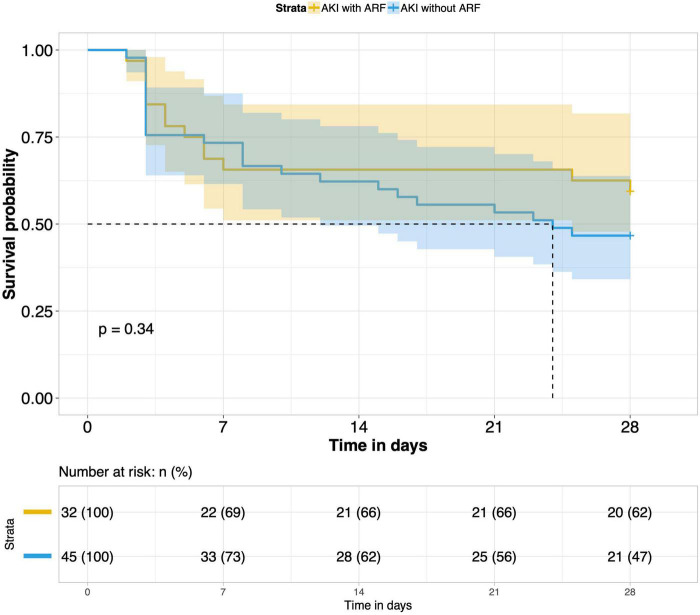
Survival analysis of AKI with ARF group and AKI without ARF group.

**FIGURE 9:**
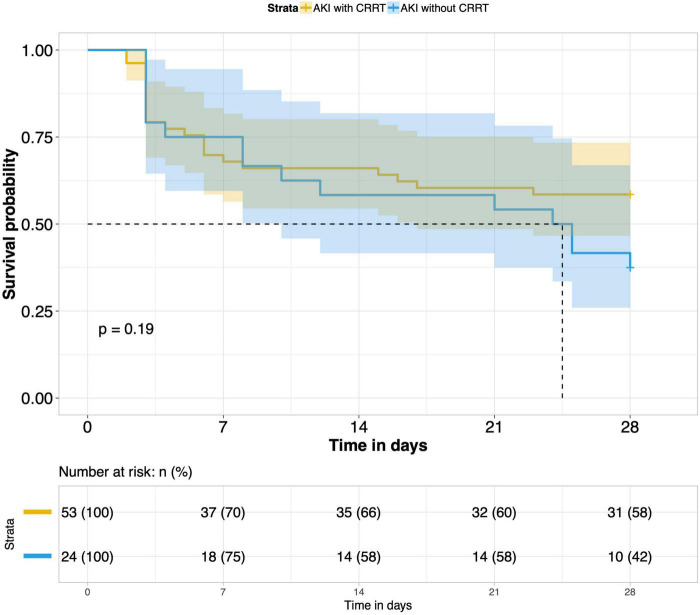
Survival analysis of AKI with CRRT and AKI without CRRT.

8. Comparison of 28-day survival rates in the two groups and in multiple subgroups of sepsis AKI: The 28-day survival rate of patients with septic AKI was significantly lower than that of patients with non-septic AKI within 28 days after ICU admission (*P* < 0.001) (Figure 5). In the three subgroups of septic AKI (septic AKI combined with septic shock, septic cardiac dysfunction or acute respiratory failure), the 28-day survival rate of septic AKI combined with septic shock decreased significantly (*P* = 0.005) (Figure 6); However, there was no significant difference in the other two subgroups of patients (*P* = 0.07, *P* = 0.34) (Figures 7, 8).

9. Effects of CRRT treatment on the short-term prognosis of septic AKI patients: According to whether CRRT was performed in the ICU, patients with septic AKI were divided into the CRRT group and the non-CRRT group. There was not any significant difference in the 28-day outcome of the two groups. CRRT had no meaningful effect on the short-term prognosis of septic AKI patients (*P* = 0.19) (Figure 9).

## Discussion

Septic AKI is a life-threatening complication characterized by an abrupt deterioration in renal function, manifested as elevated serum creatinine levels, oliguria, or both. It closely relates to infection or sepsis. Septic AKI is one of the earliest focal manifestations in patients with sepsis. Current estimates suggest that septic AKI affects 10–67% of patients with sepsis (8, 15). However, more than two-thirds of patients with septic shock may be complicated with septic AKI (16). For unexplained AKI, the possibility of sepsis should be examined first. Cancer patients are more likely to suffer from sepsis and have a significantly higher mortality rate due to sepsis than non-cancer patients (14). Our study aimed to understand the related factors of septic AKI in cancer patients with sepsis and is used as a basis for the prevention, treatment and renal function recovery of septic AKI for this population.

We found that there may be a definite relationship between septic AKI and cancer type. Regroup analysis showed that sepsis patients with retroperitoneal and urinary tumors were more vulnerable to septic AKI. For the two types of cancer patients, we analyzed the reasons. The mechanism of retroperitoneal and urinary tumors with septic acute kidney injury may include the following: Firstly, the tumor has oppressed or invaded the urinary system, causing local obstruction or postrenal obstruction, resulting in impaired renal function. Secondly, most patients with retroperitoneal and urinary tumors have undergone surgery, and there is a risk of low organ perfusion during the operation. Some patients may undergo single nephrectomy, and patients may be complicated with abdominal infection, paralytic intestinal obstruction, intra-abdominal hypertension after surgery (17). In addition, tumor-related thrombotic microvascular disease and septic coagulation dysfunction may affect the kidneys, resulting in acute kidney damage caused by renal microvascular thrombosis with endothelial swelling and microvascular obstruction (18). All of the above related factors may significantly increase the probability of septic AKI in these cancer patients. However, it was not found that the two types of cancers were closely related to the occurrence of septic AKI in the septic patients with the two types of cancers were included in the multivariate analysis.

Our study also concluded that lactate, SOFA score and septic shock were closely related to the occurrence of septic AKI with LASSO regression and Logistic regression. Serum lactate levels in the septic AKI group were significantly higher than those in the non-septic AKI group. The serum lactate level is a sensitive but non-specific indicator of metabolic stress (19). As a product of anaerobic glycolysis, lactate is markedly elevated in settings of hypoxia, stress, and critical illness (20). Most studies have demonstrated that high levels of lactate are significantly positively correlated with sepsis mortality, and the higher the lactate level is, the worse the prognosis of sepsis (21, 22). Hyperlactatemia is a significant manifestation of increasing tissue anaerobic metabolism in patients with sepsis. It is regarded as a sensitive marker of systemic or local organ tissue hypoperfusion (23). Based on the above studies, it is reasonable to believe that elevated lactate levels can predict renal hypoperfusion, which may eventually progress to AKI. SOFA score in the septic AKI group was also significantly higher than that in the non-septic AKI group. The SOFA score is a key component of the third edition of the definition of sepsis. Clinical diagnosis of infection and SOFA ≥ 2 points can be considered as the definition of sepsis (24). The higher the SOFA score, the more severe organ dysfunction due to sepsis. In our study, the differences in SOFA score between the two groups were consistent with the short-term prognosis, which suggested that the higher the SOFA score, the more severe the illness and the worse the prognosis. Studies have demonstrated that there is a good correlation between the SOFA score and lactate level. The higher the SOFA score is, the higher the lactate level in serum, both of which are signals of increased organ dysfunction and suggest the need for urgent medical intervention (25). We also found that the proportion of patients with septic shock in the septic AKI group was considerably higher than that in the non-septic AKI group. This indicated that septic shock was closely related to the occurrence of septic AKI, which was an independent risk factor for septic AKI. Septic shock leads to systemic hypotension and hypoperfusion of multiple organs, including kidney hypoperfusion. In addition, studies have shown that septic shock may lead to dysfunction of the renal vascular bed, leading to a dramatic decrease in GFR and the development of septic AKI (26). Finally, we carried out a bivariate correlation analysis of these three variables, which showed a significant positive correlation among these variables (*P* < 0.001). This result shows that septic shock may have higher levels of blood lactate, and both of which are positively correlated with the severity of the disease, that is, SOFA score.

In our study, rates of MV and CRRT for the septic AKI group were significantly higher than those of the non-septic AKI group, and the MV time and the ICU stay time were also significantly prolonged. There was a major difference in the 28-day mortality between the above two groups. The 28-day mortality was also significantly increased when septic AKI was combined with septic shock. We compared the effect of CRRT on the prognosis of patients with septic AKI. CRRT cannot prolong the short-term survival time of patients with septic AKI, so CRRT did not improve the short-term prognosis of septic AKI. These conclusions are in agreement with most studies (27, 28).

Our study on septic AKI is of definite clinical significance. Firstly, the group of this study focused on cancer patients with sepsis, and we found that septic cancer patients of retroperitoneal and urinary tumors were more likely to have septic AKI. The group studied and this conclusion are not common in previous studies. Secondly, we screened out three variables with the intersection of Wayne diagram adopted from the combination of Lasso regression and logistic regression. The prediction of the combined ROC based on the three variables for the occurrence of septic AKI has good performance. Later, it can be modeled and verified after increasing the sample size. If the predictive ability of the model is reliable, it can be adopted in clinical application to judge the prognosis of septic AKI at an early stage. Finally, we understand that if cancer patients with sepsis have septic AKI at the same time, the short-term outcome will be poor, and CRRT cannot effectively improve the prognosis. The above aspects are helpful for us to understand the risk factors of septic AKI in cancer patients with sepsis, which play a good reference role in the diagnosis, treatment, and prognosis of septic AKI in cancer patients.

However, our study has its limitations. Firstly, this study was a retrospective study, and our data were taken from single-center studies, so the incidence and severity of septic AKI may be biased. Secondly, for all patients with septic AKI, we focused on the short-term outcomes within 28 days after ICU admission and lacked 90-day or longer follow-up data on cancer patients with sepsis. The lack of awareness of the long-term survival and physical and mental health of patients with sepsis is also something that needs to be improved in future research. In addition, in view of the small number of CRRT treatments, a total of 24 cases, we did not conduct a subgroup analysis, but only to explore the overall prognostic differences. If patients with septic AKI were graded to different subgroups according to the KIDGO criteria, and the prognostic value of CRRT in each subgroup is compared, different results may be obtained, which also represents one of the limitations of this study.

## Conclusion

Lactate level, SOFA score and septic shock were closely related to the occurrence of septic AKI in the ICU. The clinical outcomes within 28 days after ICU admission of cancer patients with septic AKI were worse than those without septic AKI. The short-term outcome was worse in patients with septic AKI complicated with septic shock. CRRT does not have any significant effect on the short-term prognosis of cancer patients with septic AKI in the ICU. This study was a preliminary exploration of the incidence, influencing factors and clinical outcomes of septic AKI in cancer patients with sepsis, which has certain guiding significance for the diagnosis, treatment and prognosis of septic AKI.

## Data availability statement

The raw data supporting the conclusions of this article will be made available by the authors for a reasonable purpose, without undue reservation.

## Ethics statement

The study was approved by the Ethics Committee of Peking University Cancer Hospital and all patients provided written informed consent for the treatment of sepsis and related scientific research purposes.

## Author contributions

YY and JD designed, analyzed, and drafted the manuscript. XC and RC collected and interpreted the patients’ data. HW administered and revised the manuscript. All authors read and approved the final manuscript.
